# Supplementary material for: Therapeutic Potential of a Novel Vitamin D3 Oxime Analogue, VD1-6, with CYP24A1 Enzyme Inhibitory Activity and Negligible Vitamin D Receptor Binding
Source: Biomolecules. 2022 Jul 8;12(7):960. doi: 10.3390/biom12070960 (PMC9312876; doi:10.3390/biom12070960)
Supplement: Supplementary file 1 [file biomolecules-12-00960-s001.zip › biomolecules-1749681-supplementary.pdf]

Therapeutic Potential of a Novel Vitamin D_3 Oxime Analogue, VD1-6, with CYP24A1 Enzyme Inhibitory Activity and Negligible Vitamin D Receptor Binding

Ali K. Alshabrawy [1,2,†], Yingjie Cui [3,4,†], Cyan Sylvester [1], Dongqing Yang [5], Emilio S. Petito [1], Kate R. Barratt [1], Rebecca K. Sawyer [1], Jessica K. Heatlie [1], Ruhi Polara [1], Matthew J. Sykes [1], Gerald J. Atkins [5], Shane M. Hickey [1], Michael D. Wiese [1], Andrea M. Stringer [1], Zhaopeng Liu [4,‡] and Paul H. Anderson [1,*‡]

1 UniSA: Clinical and Health Sciences, Health and Biomedical Innovation, University of South Australia, Adelaide, SA 5001, Australia; ali.alshabrawy@mymail.unisa.edu.au (A.K.A.); cyan.sylvester@mymail.unisa.edu.au (C.S.); emilio.petito@mymail.unisa.edu.au (E.S.P.); kate.barratt@unisa.edu.au (K.R.B.); rebecca.sawyer@unisa.edu.au (R.K.S.); jessica_kate.heatlie@mymail.unisa.edu.au (J.K.H.); polra001@mymail.unisa.edu.au (R.P.); matt.sykes@unisa.edu.au (M.J.S.); shane.hickey@unisa.edu.au (S.M.H.); michael.wiese@unisa.edu.au (M.D.W.); andrea.stringer@unisa.edu.au (A.M.S.)

2 Pharmaceutical Chemistry Department, Faculty of Pharmacy, Helwan University, Cairo 11795, Egypt

3 Department of Medicinal Chemistry, Key Laboratory of Chemical Biology (Ministry of Education), School of Pharmaceutical Sciences, Cheeloo College of Medicine, Shandong University, Jinan 250012, China; yingjiecui@sina.cn

4 Department of Pharmacy, Shandong Provincial Hospital Affiliated to Shandong First Medical University, Jinan 250021, China; liuzhaop@sdu.edu.cn

5 Centre for Orthopaedic and Trauma Research, Faculty of Health and Medical Sciences, The University of Adelaide, Adelaide, SA 5005, Australia; dongqing.yang@adelaide.edu.au (D.Y.); gerald.atkins@adelaide.edu.au (G.J.A.)

* Correspondence: paul.anderson@unisa.edu.au

† These authors contributed equally to this work.

‡ Co-senior authors contributed equally.

CONTENTS

- Reagents, chemicals and apparatus used in chemical synthesis and purification p 2
- Synthetic protocols p 2
- ^{1}H and ^{13}C NMR and ESI MS spectra for compounds **VD1-2 – VD1-4** and **VD1-6** p 5
- Chromatograms of 1,25(OH)$_2$D$_3$-DAPTAD and 1,25(OH)$_2$D$_3$-*d6*-DAPTAD derivatives p 17
- Linearity, accuracy and precision testing for 1,25(OH)$_2$D$_3$ LC-MS/MS analysis p 18

Reagents, chemicals and apparatus used in chemical synthesis and purification

Anhydrous tetrahydrofuran (THF) was purchased from Sigma-Aldrich (Shanghai). All other reagents used in the synthesis of **VD1-6** (mentioned under S.2), including **VD1-5** and solvents were purchased from Sinopharm Chemical Reagent Co., Ltd (Shanghai). ^{1}H and ^{13}C NMR spectra were recorded at room temperature on a Bruker 400 MHz NMR or a Bruker 600 MHz NMR spectrometer in $CDCl_3$ as the solvent. MS spectral data were acquired by a Sciex® API 4000 mass spectrometer. Column chromatography was performed on silica gel (200–300 mesh)

Synthetic protocols

(6*R*)-2,2-Dimethyl-6-((1*R*,4*S*,7a*R*)-7a-methyl-4-((triethylsilyl)oxy)octahydro-1*H*-inden-1-yl)heptan-3-one (VD1-2)

A *n*-BuLi (1.09 mL, 2.5 M in *n*-hexane) solution was added slowly into diisopropylamine (0.27 mL, 2.22 mmol) at 0 ℃ under a nitrogen atmosphere. The mixture was diluted using THF (5 mL) at −78 ℃ and stirred for 20 min. 3,3-Dimethyl-2-butanone (0.37 mL, 2.96 mmol) was then added and stirred for 30 min, before HMPA (1.71 mL, 7.74 mmol) was added and stirred for a further 15 min. To the stirring mixture was added iodide **VD1-1** (130 mg, 0.31 mmol) and the stirring was maintained for 2 h before being warmed to room temperature. The reaction was quenched with H_2O (1.0 mL) and extracted with EtOAc (3 × 20 mL). The organic portions were combined and washed with brine (10 mL), dried over anhydrous Na_2SO_4, and concentrated. The crude material was purified using column chromatography (*n*-hexane) to give **VD1-2** (85 mg, 72%) as a colourless oil. ^{1}H NMR (400 MHz, $CDCl_3$) δ 4.03 (s, 1H), 2.54–2.33 (m, 2H), 1.99–1.90 (m, 1H), 1.87–1.76 (m, 2H), 1.75–1.63 (m, 2H), 1.59–1.50 (m, 1H), 1.43–1.18 (m, 9H), 1.13 (s, 9H), 0.99–0.84 (m, 15H), 0.54 (q, *J* = 8.0 Hz, 6H); ^{13}C NMR (100 MHz, $CDCl_3$) δ 216.8, 56.8, 53.2, 44.4, 42.3, 40.9, 35.2, 34.8, 33.5, 30.2, 27.4, 26.6, 23.2, 18.6, 17.8, 13.7, 7.1, 5.1. MS (ESI) calc. for $C_{25}H_{48}NaO_2Si$ $[M+Na]^+$: 431.33, found: 431.43, 839.21 $[2M+Na]^+$.

(6*R*,*E*)-2,2-Dimethyl-6-((1*R*,4*S*,7a*R*)-7a-methyl-4-((triethylsilyl)oxy)octahydro-1*H*-inden-1-yl)heptan-3-on-*O*-methyl oxime (VD1-3)

To a solution of **VD1-2** (300 mg, 0.73 mmol) in pyridine (6 mL) was added NH_2OCH_3·HCl (1.23 g, 14.70 mmol). The mixture was stirred at room temperature for 4 h before being quenched with H_2O (10 mL) and extracted with EtOAc (3 × 30 mL). The organic portions were combined and washed with brine (20 mL), dried over anhydrous Na_2SO_4, and concentrated. The crude material was purified using column chromatography (2–10% CH_2Cl_2 in *n*-hexane) to give **VD1-3** (220 mg, 69%) as a colourless oil. ^{1}H NMR (400 MHz, $CDCl_3$) δ 4.03 (s, 1H), 3.78 (s, 3H), 2.38–2.25 (m, 1H), 2.08–1.89 (m, 2H), 1.91–1.74 (m, 2H), 1.72–1.18 (m, 12H), 1.10 (s, 9H), 1.00–0.90 (m, 15H), 0.55 (q, *J* = 8.0 Hz, 6H); ^{13}C NMR (100 MHz, $CDCl_3$) δ 167.6, 69.6, 61.1, 56.5, 53.5, 42.3, 41.0, 37.4, 36.5, 34.8, 32.6, 28.0, 27.4, 23.4, 23.3, 18.5, 17.9, 13.7, 7.1, 5.1. MS (ESI) calc. for $C_{26}H_{52}O_2Si$ $[M+H]^+$: 438.38, found: 438.62.

(1*R*,7a*R*)-1-((*R*,*E*)-5-(Methoxyimino)-6,6-dimethylheptan-2-yl)-7a-methyloctahydro-4*H*-inden-4-one (VD1-4)

To a solution of **VD1-3** (220 mg, 0.50 mmol) in MeOH (4 mL) was added CSA (151 mg, 0.65 mmol). The mixture was stirred at room temperature for 1 h before the reaction was quenched with sat. $NaHCO_3$ (5 mL). The mixture was extracted with EtOAc (3 × 20 mL) and the organic portions were combined and washed with brine (20 mL), dried over anhydrous Na_2SO_4, and concentrated. The crude material was dissolved in CH_2Cl_2 (5 mL) and PDC (415 mg, 0.32 mmol) was added. The mixture was stirred at room temperature for 40 min before being diluted with CH_2Cl_2 (20 mL) and filtered through celite. The filtrate was collected and washed with sat, $NaHCO_3$ (10 mL) and brine (10 mL), dried over anhydrous Na_2SO_4, and concentrated. The crude material was purified using column chromatography (5% EtOAc in *n*-hexane) to give **VD1-4** (110 mg, 68%) as a colourless oil. ^{1}H NMR (400 MHz, $CDCl_3$) δ 3.78 (s, 3H), 2.47–2.42 (m, 1H), 2.34–2.20 (m, 3H), 2.12–2.09 (m, 1H), 2.06–1.98 (m, 2H), 1.96–1.87 (m, 2H), 1.77–1.69 (m, 1H), 1.57–1.49 (m, 3H), 1.47–1.40 (m, 2H), 1.36–1.29 (m, 2H), 1.10 (s, 9H), 1.01 (d, *J* = 6.0 Hz, 3H), 0.64 (s, 3H); ^{13}C NMR (100 MHz, $CDCl_3$) δ 212.1, 167.1, 62.1, 61.1, 56.3, 50.0, 41.1,

39.1, 37.4, 36.6, 32.5, 28.0, 27.6, 24.2, 23.2, 19.3, 18.6, 12.6. MS (ESI) calc. for $C_{20}H_{36}NO_2$ $[M+H]^+$: 322.27, found: 322.76.

(6*R*,*E*)-6-((1*R*,7a*R*,*E*)-4-((*Z*)-2-((3S,5R)-3,5-dihydroxy-2-methylenecyclohexylidene)ethylidene)-7a-methyloctahydro-1*H*-inden-1-yl)-2,2-dimethylheptan-3-on-*O*-methyl oxime （VD1-6）

To a stirring solution of **VD1-5** (286 mg, 0.49 mmol) in anhydrous THF (6 mL) at −78 °C under nitrogen atmosphere was added *n*-BuLi (0.2 mL, 0.50 mmol, 2.5 M in *n*-hexane) dropwise. The mixture was stirred at −78 °C for 15 min before a solution of **VD1-4** (110 mg, 0.34 mmol) in anhydrous THF (3 mL) was added. The stirring was continued at −78 ℃ for 4 h and then 12 h at 0 ℃. The reaction was quenched with sat. NH_4Cl (1 mL) and extracted with EtOAc (3 × 20 mL). The organic portions were combined and washed with brine (10 mL), dried over anhydrous Na_2SO_4, and concentrated. The crude material was purified using column chromatography (1% EtOAc in *n*-hexane) to give a colourless oil (132 mg, 56.6%). A portion of the oil (60 mg, 0.087 mmol) was dissolved in MeOH (3 mL). To this solution, CSA (60 mg, 0.26 mmol) was added, and the mixture was stirred at room temperature for 3 h before being quenched with sat. $NaHCO_3$ (2 mL). The mixture was extracted with EtOAc (3 × 10 mL) and the organic portions were combined and washed with brine (5 mL), dried over anhydrous Na_2SO_4, and concentrated. The crude material was purified using column chromatography (50% EtOAc in *n*-hexane) to give **VD1-6** (30 mg, 43%, over two steps) as a colourless oil. ^{1}H NMR (600 MHz, $CDCl_3$) δ 6.39 (d, *J* = 11.2 Hz, 1H), 6.03 (d, *J* = 11.2 Hz, 1H), 5.33 (s, 1H), 5.00 (s, 1H), 4.46–4.40 (m, 1H), 4.27–4.19 (m, 1H), 3.79 (s, 1H), 2.84–2.81 (m, 1H), 2.61–2.59 (m, 3H), 2.33–2.28 (m, 2H), 2.05–1.97 (m, 4H), 1.94–1.88 (m, 2H), 1.72–1.67 (m, 2H), 1.51–1.40 (m, 6H), 1.33–1.25 (m, 5H), 1.11 (s, 9H), 0.99 (d, *J* = 6.5 Hz, 3H), 0.55 (s, 3H); ^{13}C NMR (150 MHz, $CDCl_3$) δ 167.4, 147.6, 143.3, 132.8, 125.04, 117.01, 111.8, 70.8, 66.9, 61.0, 56.3, 55.9, 45.9, 45.3, 42.9, 40.4, 37.2, 37.1, 32.5, 29.1, 27.9, 27.5, 23.6, 23.2, 18.6, 12.0. MS (ESI) calc. for $C_{29}H_{48}NO_3$ $[M+H]^+$: 458.36, found: 458.65.

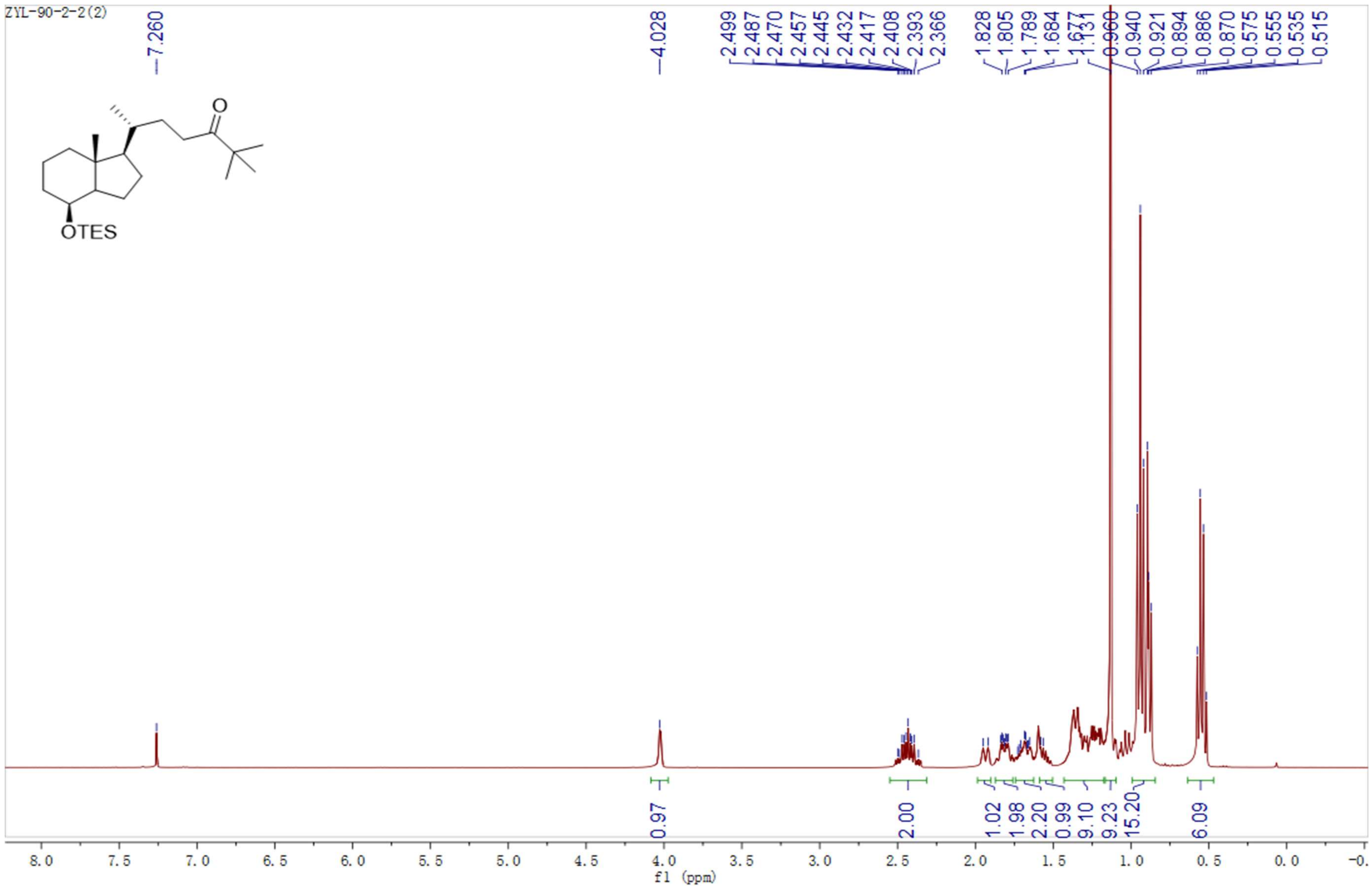

Figure S1. ^{1}H NMR spectrum of VD1-2 in $CDCl_3$.

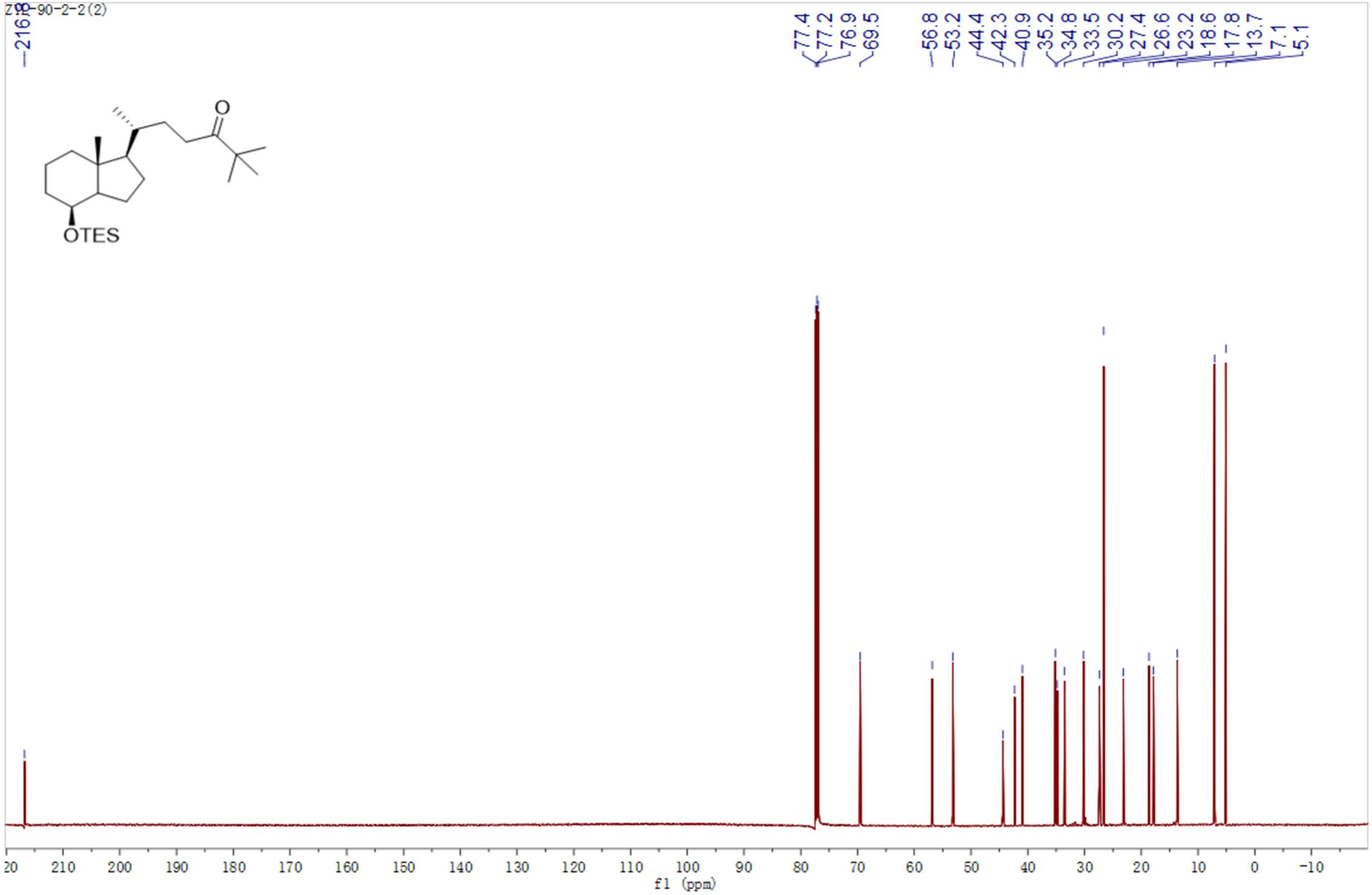

Figure S2. ^{13}C NMR spectrum of VD1-2 in $CDCl_3$.

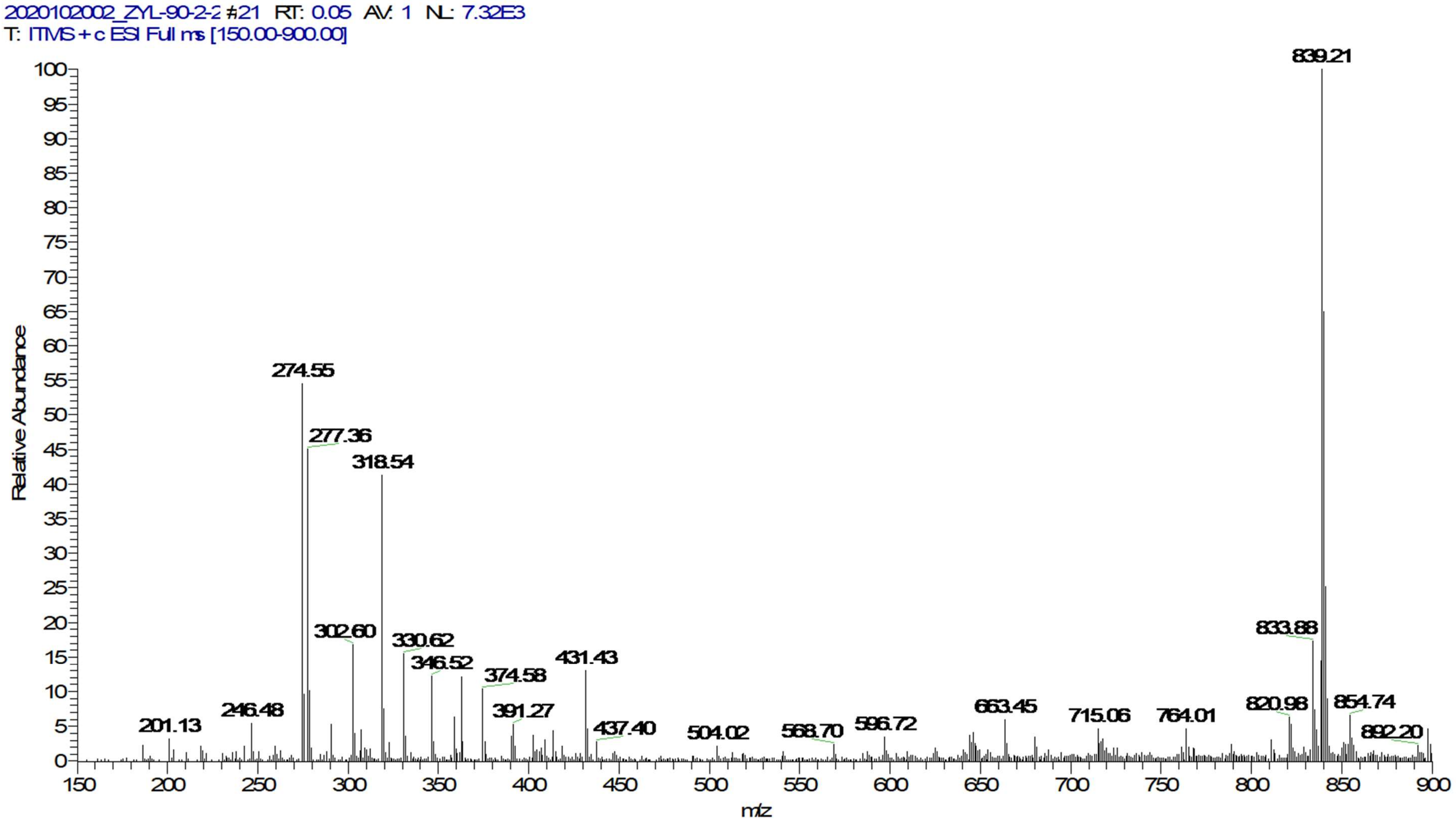

Figure S3. ESI MS spectrum of VD1-2.

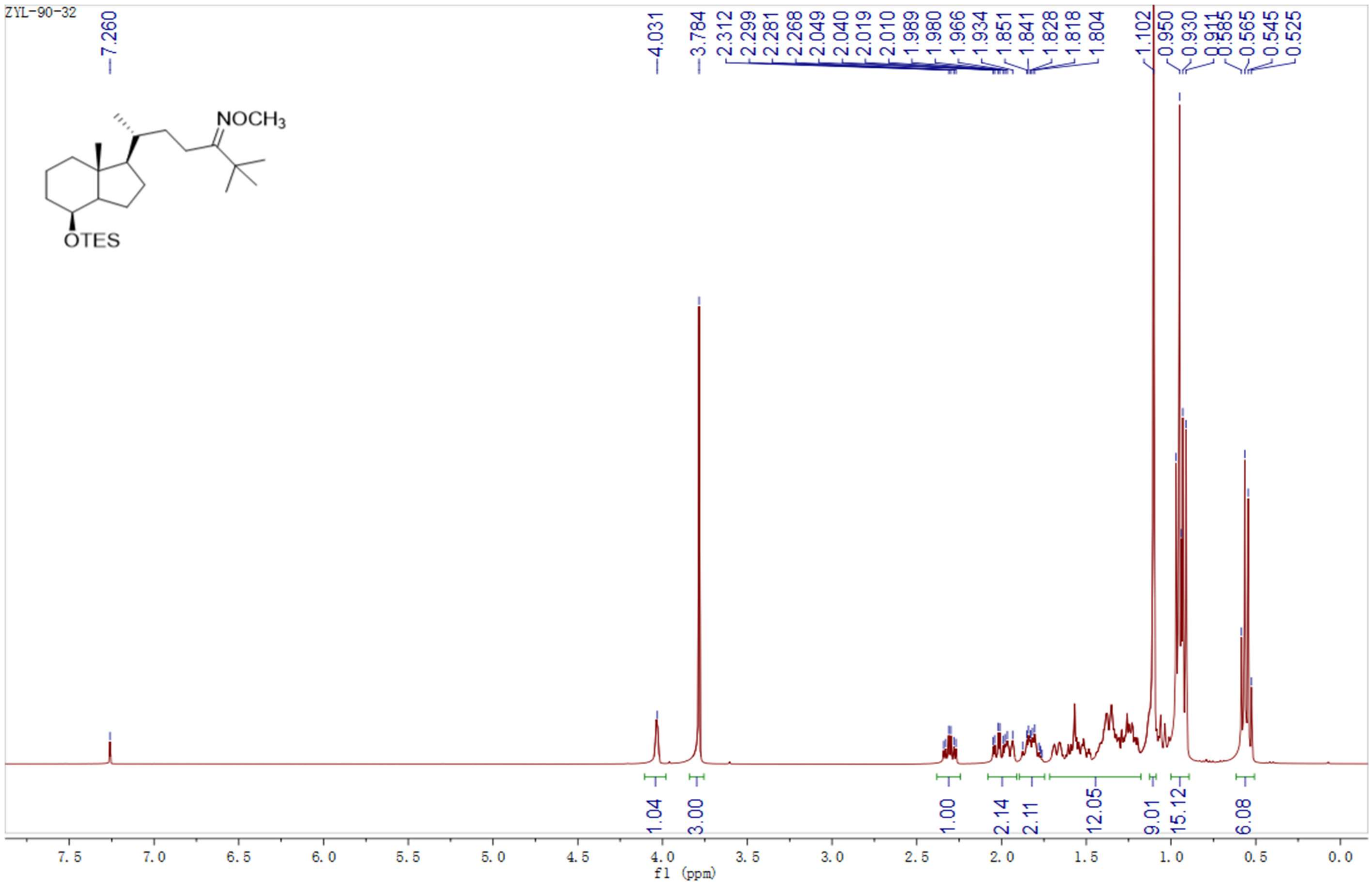

Figure S4. ^{1}H NMR spectrum of VD1-3 in $CDCl_3$.

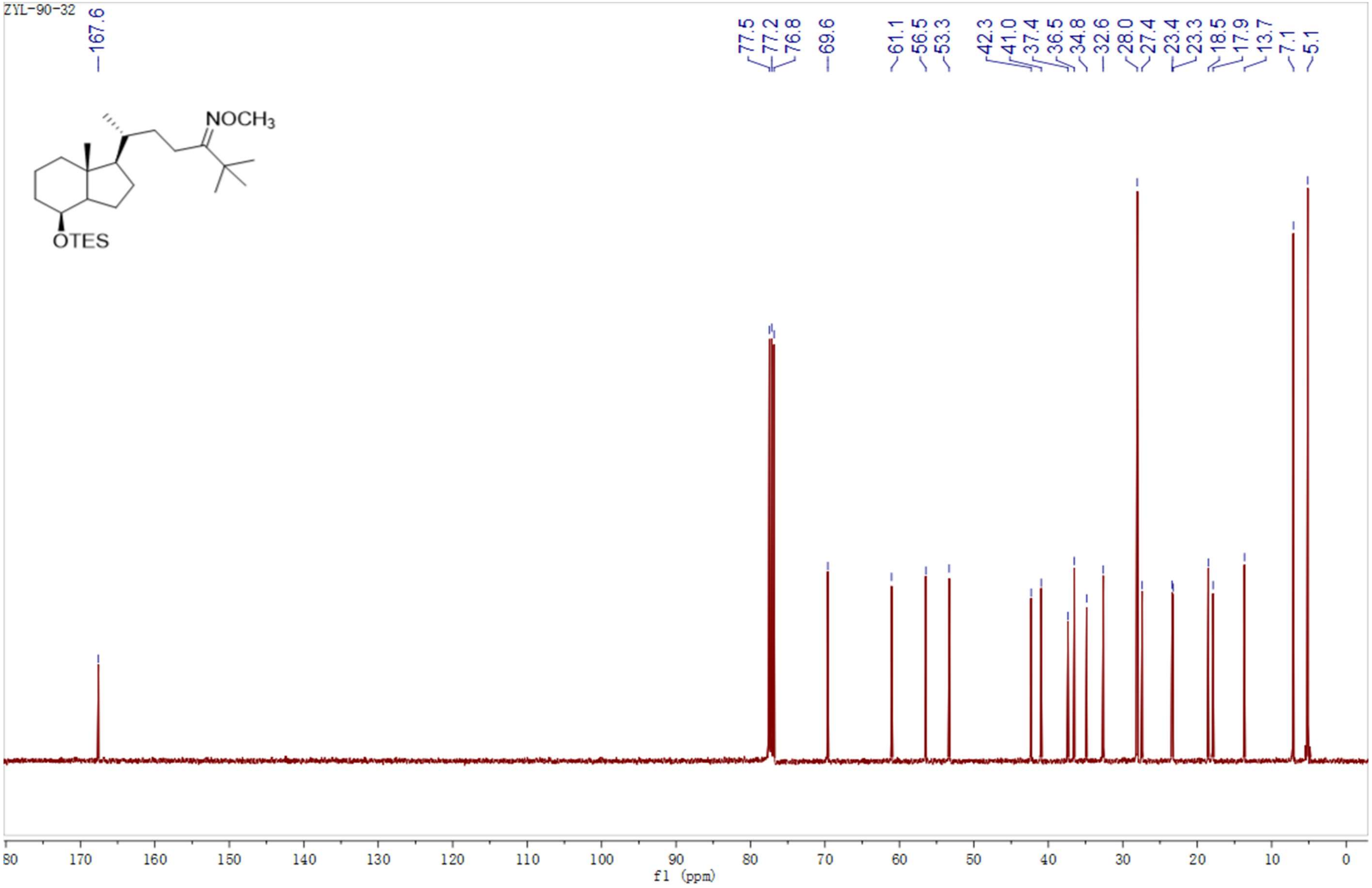

Figure S5. ^{13}C NMR spectrum of VD1-3 in $CDCl_3$.

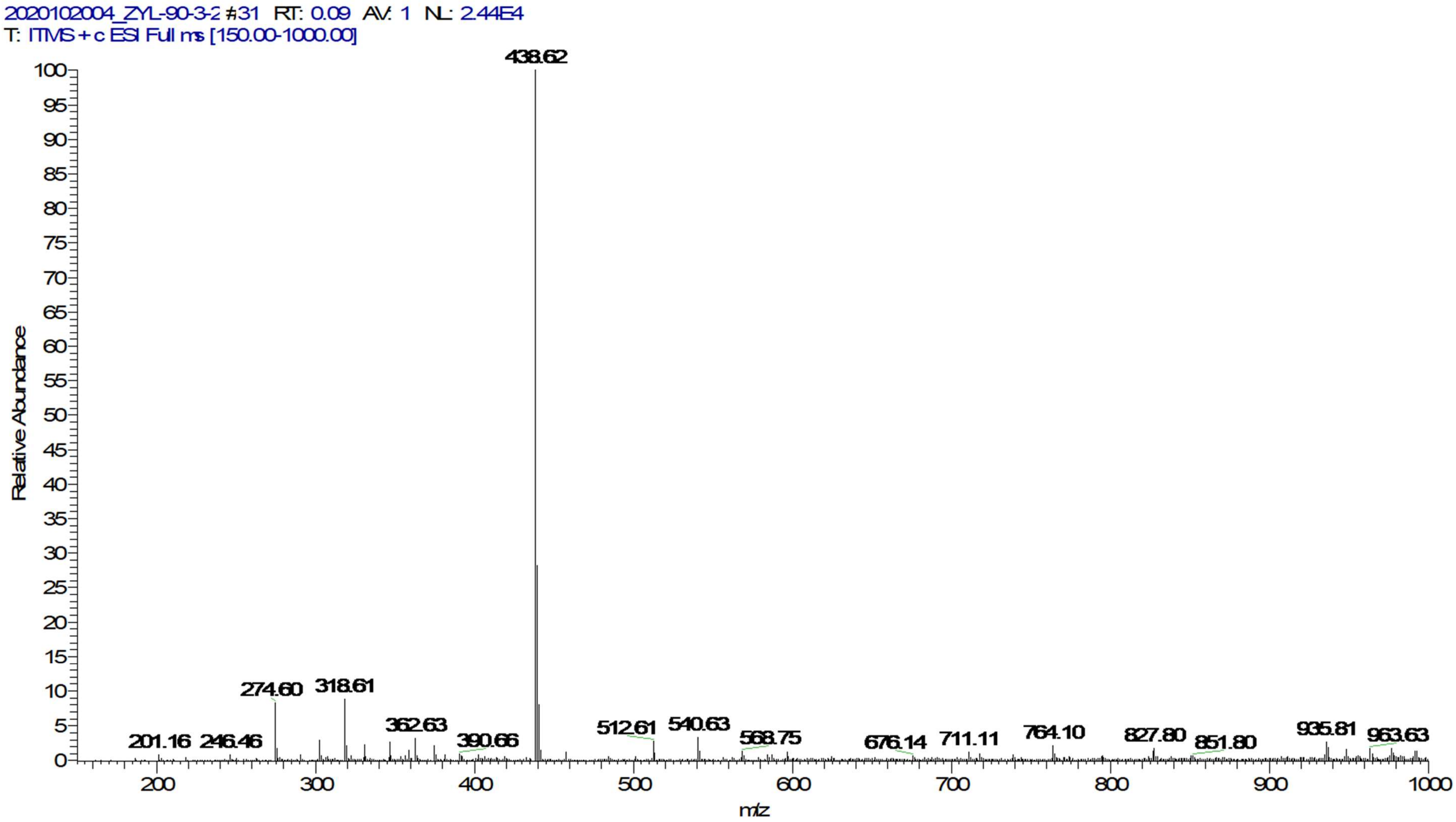

Figure S6. ESI MS spectrum of VD1-3.

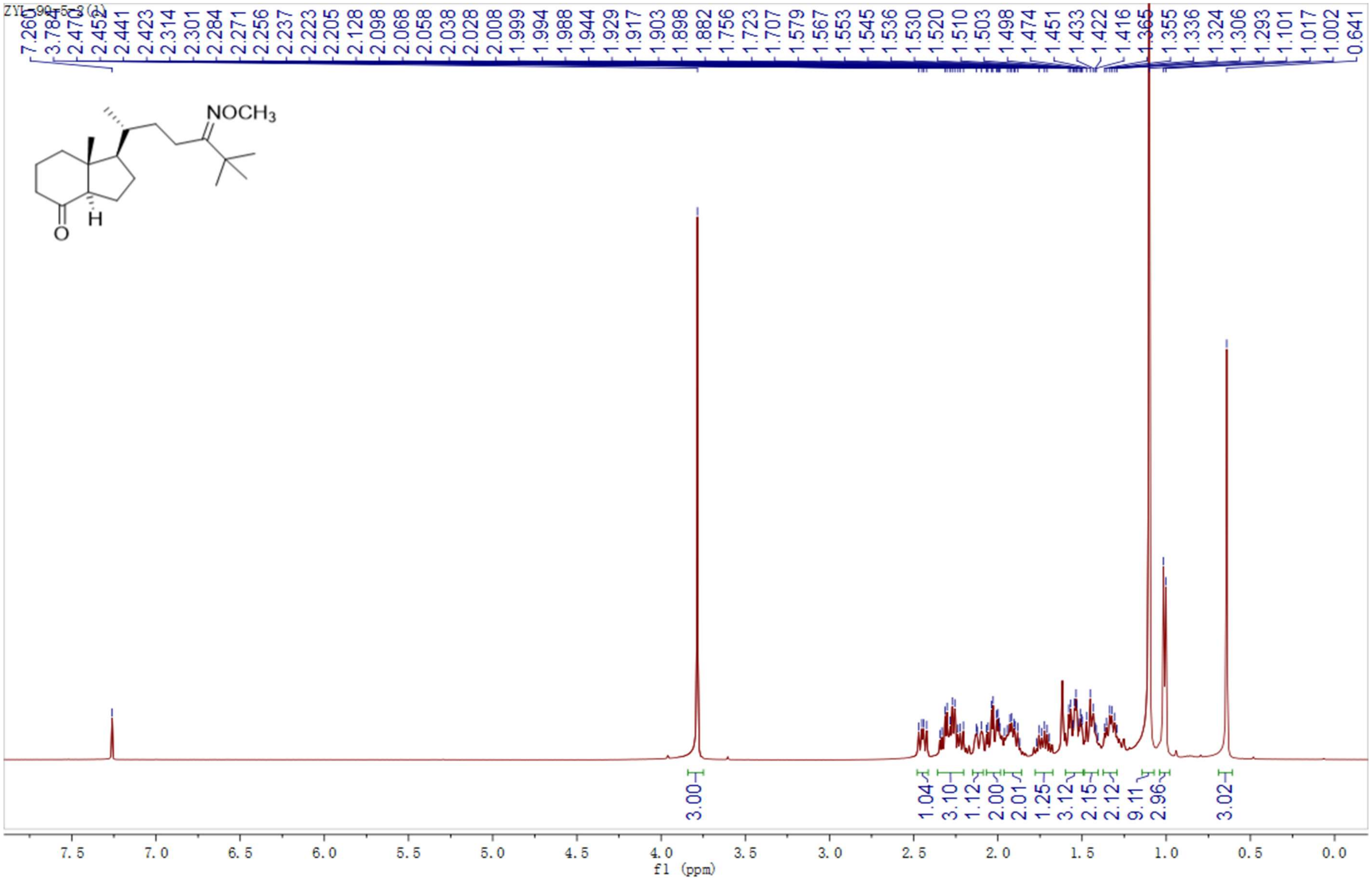

Figure S7. ^{1}H NMR spectrum of VD1-4 in $CDCl_3$.

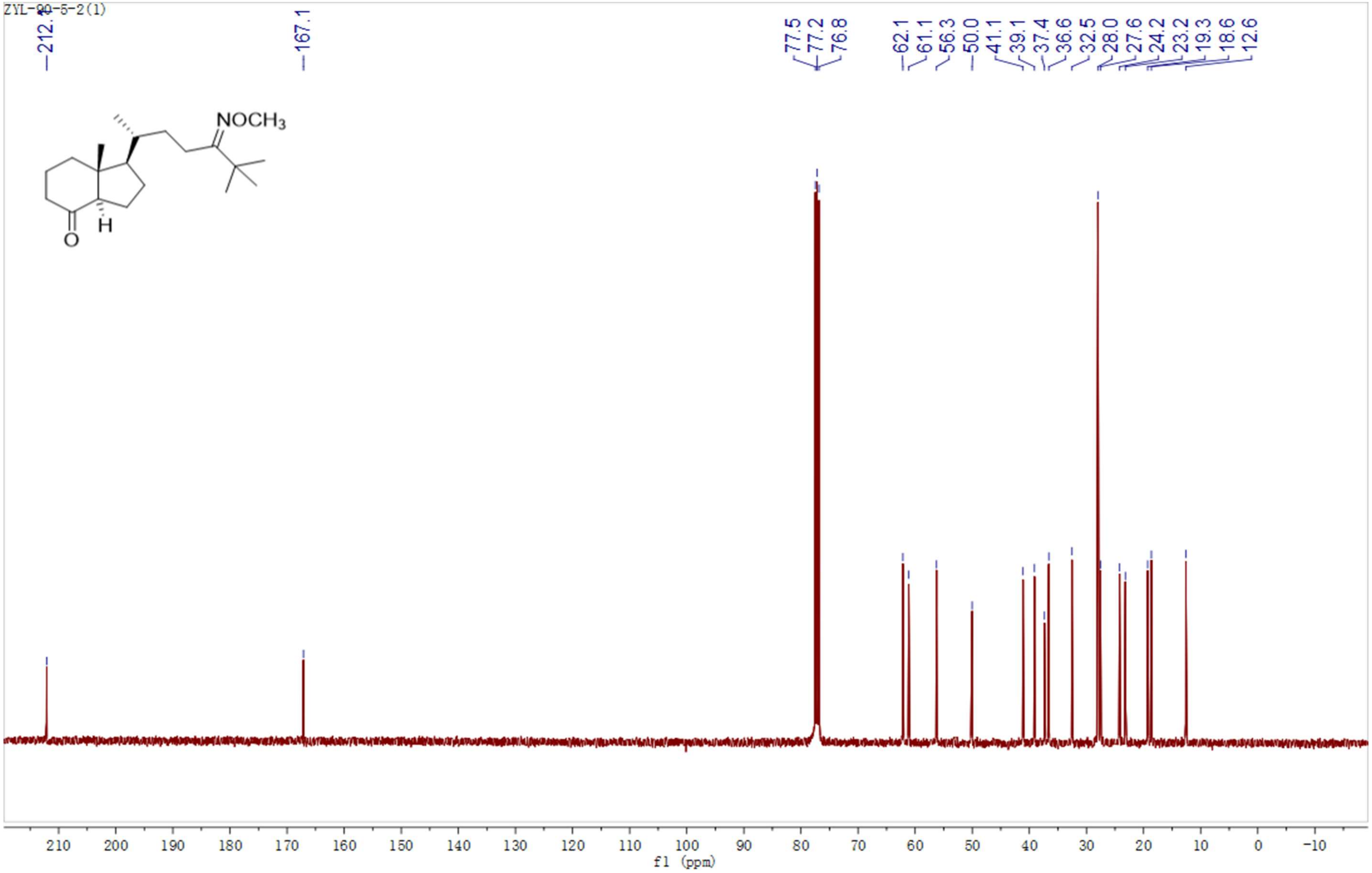

Figure S8. ^{13}C NMR spectrum of VD1-4 in $CDCl_3$.

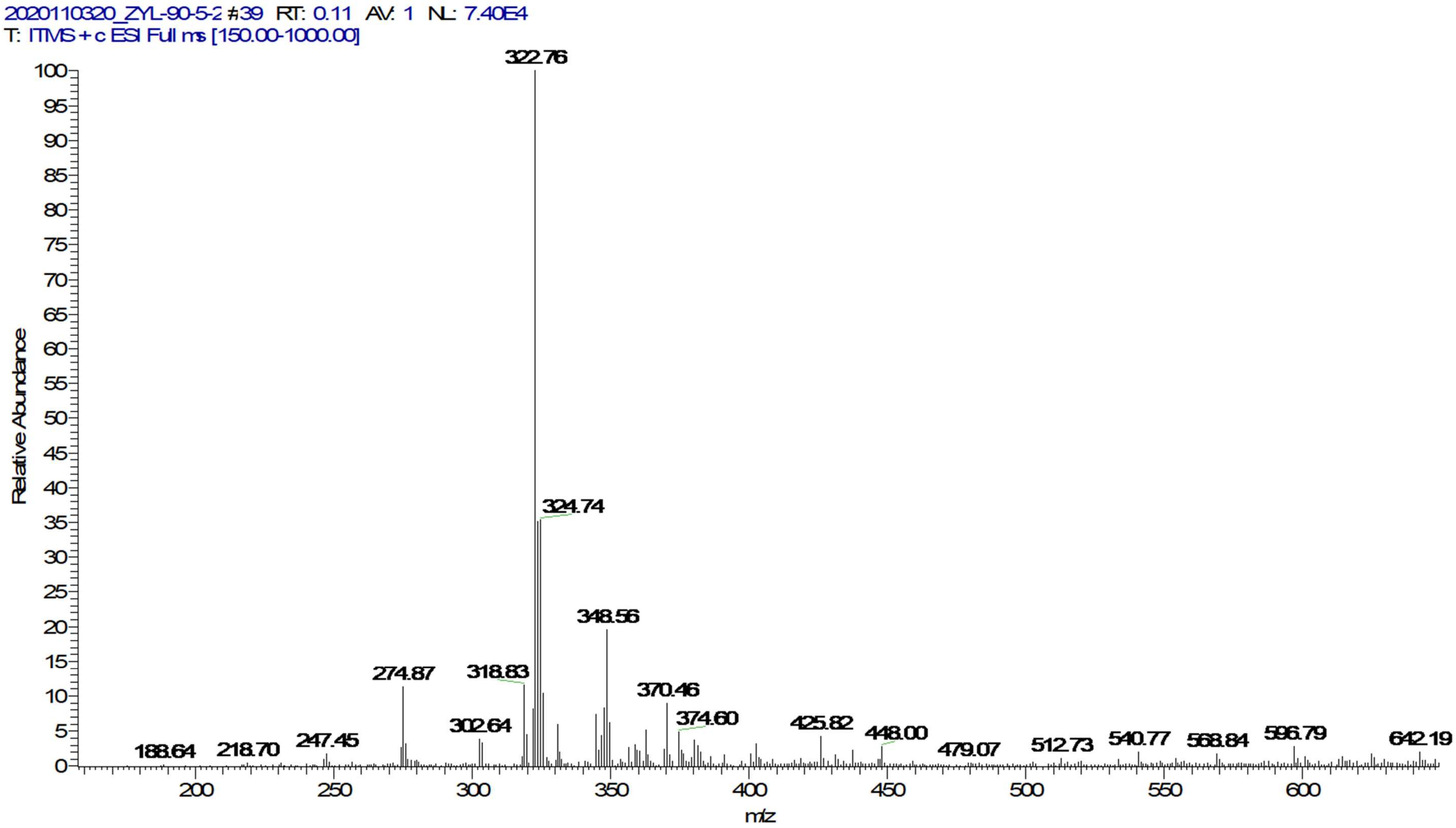

Figure S9. ESI MS spectrum of VD1-4.

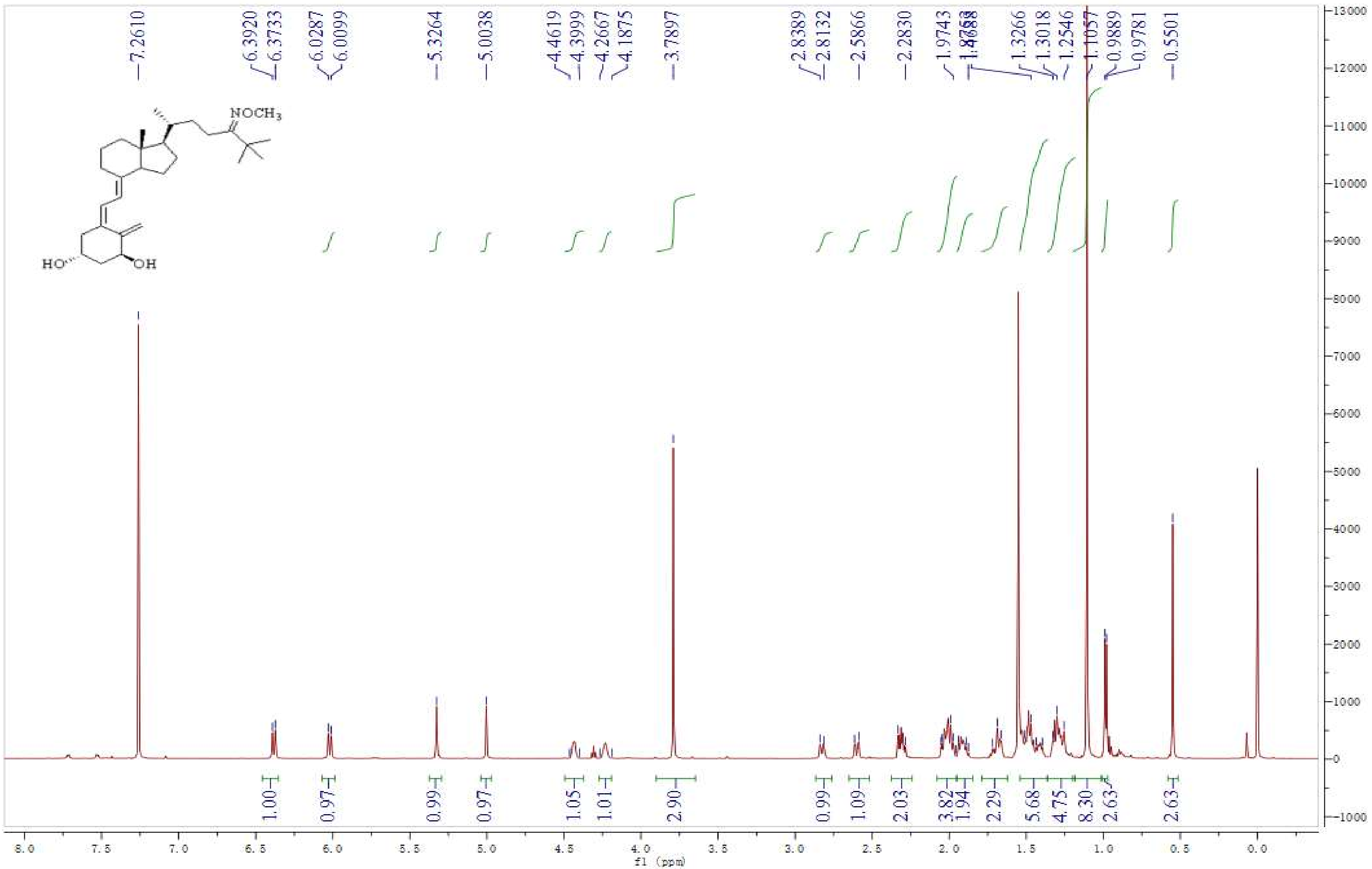

Figure S10. ^{1}H NMR spectrum of VD1-6 in $CDCl_3$.

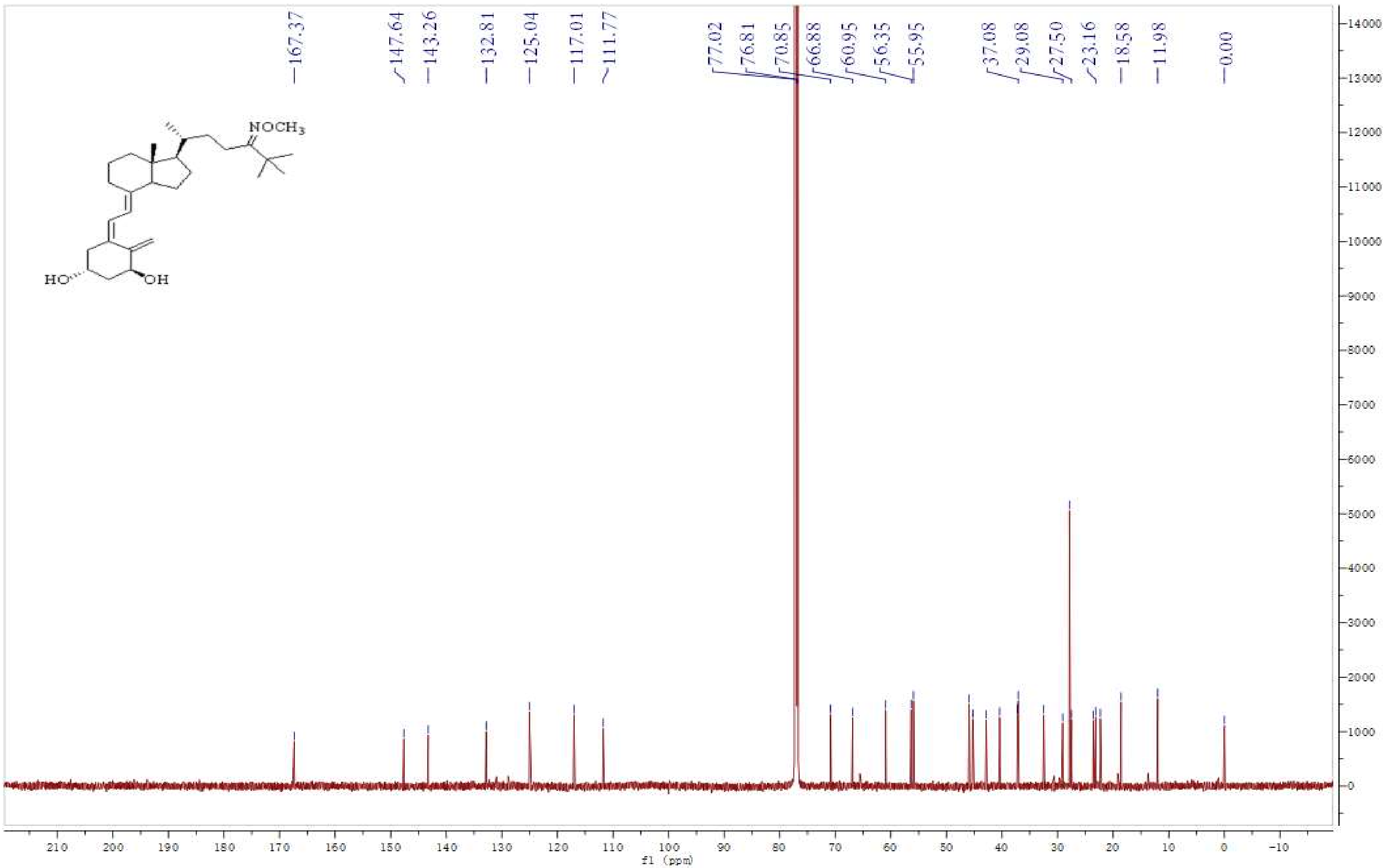

Figure S11. ^{13}C NMR spectrum of VD1-6 in $CDCl_3$.

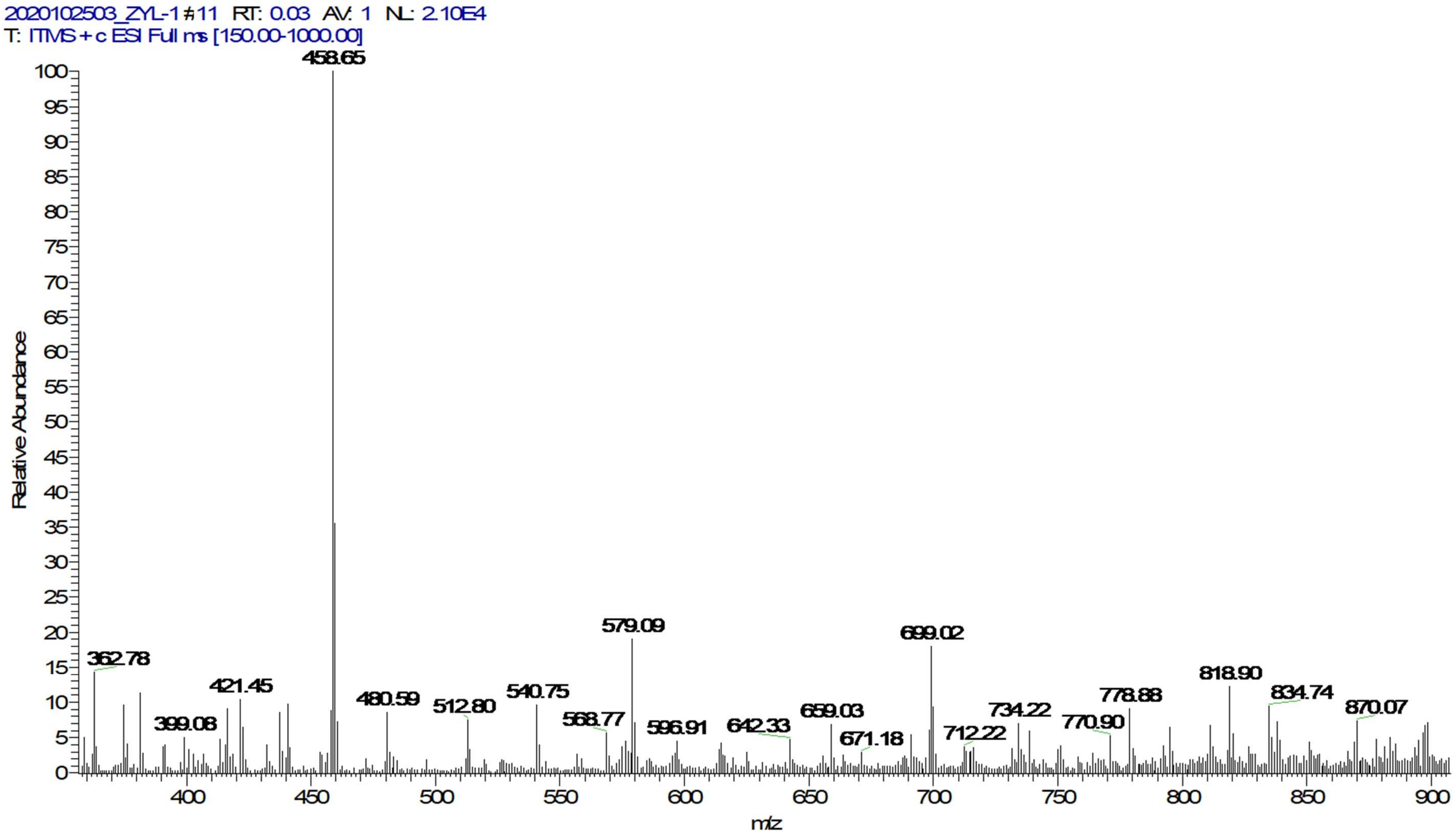

Figure S12. ESI MS spectrum of VD1-6.

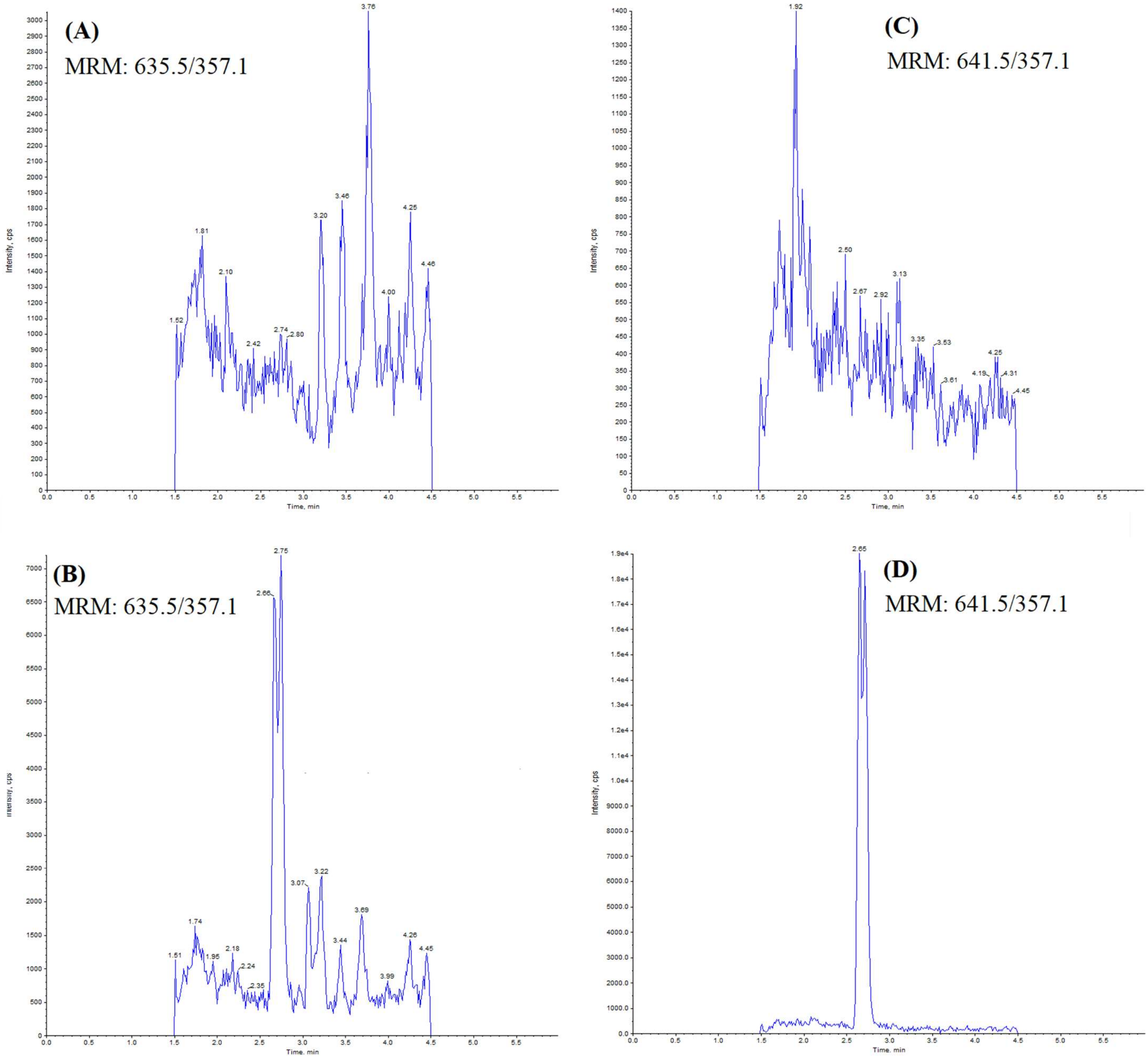

Figure S13. Chromatograms of 1,25(OH)2D3-DAPTAD samples. Illustrative chromatograms of 1,25(OH)$_2$D$_3$-DAPTAD derivative in blank (A) and LLOQ (50 pg/mL or 120 pM) (B) and of 1,25(OH)$_2$D$_3$-d_6-DAPTAD in blank (C) and at working concentration (1000 pg/mL or 2400 pM, spiking volume used 20 μL) (D).

Table S1. Reproducibility of 1,25(OH)2D3 LC-MS/MS analysis assays. Results of linearity, within-analytical batch and between analytical batches accuracy and precision testing for 1,25(OH)$_2$D$_3$ LC-MS/MS analysis

Calibration curve parameters (n = 6)		
**Linearity (pg/mL)**	***Regression coefficient**	**Range of calibrators back calculated accuracies (%)**
50-2000 pg/mL	0.9974 ± 0.0007	87.4–112

Accuracy and precision data

	Within analytical batch (n = 5)			Between analytical batches (n = 9)		
**Nominal Concentration (pg/mL)**	**Mean found (pg/mL)**	****Accuracy (%)**	**#Precision (%)**	**Mean found (pg/mL)**	****Accuracy (%)**	**#Precision (%)**
50	48.50	97.0	9.5	48.90	97.8	11.5
150	162.7	108.4	5.8	155.5	103	7.6
800	785.0	98.1	4.8	817.3	102	5.8
1600	1700	106.3	5.1	1631	102	6.9

* Mean ± Standard deviation (SD)

** % found from nominal concentration

%CV
